# A comprehensive pan-cancer analysis of CDH5 in immunological response

**DOI:** 10.3389/fimmu.2023.1239875

**Published:** 2023-09-21

**Authors:** Yuantao Li, Qikai Wu, Jiancheng Lv, Junwei Gu

**Affiliations:** ^1^ Department of Gastroenterology, Linyi County People’s Hospital, Dezhou, China; ^2^ Laboratory of Urology and Andrology, Jiangsu Clinical Medicine Research Institution, Nanjing, China; ^3^ Department of Urology, The First People's Hospital of Xiushui County, Jiujiang, Jiangxi, China

**Keywords:** cdh5, pan-cancer, prognosis, immune response, CD8 + T cells

## Abstract

**Background:**

Cadherin 5 (CDH5) functions critically in maintaining cell adhesion and integrity of endothelial and vascular cells. The expression of CDH5 is abnormal in tumor cells, which may have great potential to serve as a new immune checkpoint. The current pan-cancer analysis was performed to better understand the role of CDH5 in tumor.

**Methods:**

The clinical significance and immunological function of CDH5 in pan-cancers were comprehensively analyzed based on the correlations between CDH5 and clinicopathologic features, prognosis values, tumor mutation burden (TMB), microsatellite instability (MSI), immune cells infiltration and immune response genes using 33 datasets from The Cancer Genome Atlas (TCGA). We further confirmed the expression of CDH5 in bladder cancer (BCa) tissues and cell lines. The CD8^+^ T cells were screened from peripheral blood of healthy controls and activated. BCa cell-CD8^+^ T cell co-culture assay and ELISA assay were carried out to verify the immunological function of CDH5.

**Results:**

The expression of CDH5 was down-regulated in 8 types of tumors including in BCa but up-regulated in 4 types of tumors. CDH5 was significantly correlated with tumor stage in 6 types of tumors. In addition, CDH5 was positively or negatively correlated with tumor prognosis. Furthermore, CDH5 was closely associated with TMB in 15 types of tumors and with MSI in 9 types of tumors. KEGG-GSEA and Hallmarks-GSEA analyses results indicated that CDH5 was positively related to immune response in most tumor types. In many tumors, CDH5 showed a positive correlation with immune cell infiltration. Enrichment analyses demonstrated that CDH5 was significantly related to the expression of many immunomodulators and chemokines. Further experiments showed that CDH5 was low-expressed in BCa tissues and cell lines in comparison to adjacent normal tissues and normal urothelial cell line, but it was positively associated with a better prognosis of BCa patients. The results of *in vitro* co-culture assay and ELISA assay demonstrated that CDH5 could promote the function of CD8^+^ T cells in TME of BCa.

**Conclusion:**

In summary, CDH5 was positively associated with a favorable prognosis and effective immune response in tumors, showing a great potential to serve as a novel tumor biomarker and immune checkpoint.

## Introduction

Cancer is one of the major causes leading to death worldwide, and it also seriously affects the physical and mental health of patients, resulting in a significant decline in patients’ life quality (1, 2). In 2022, the United States has reported more than 1.9 million new cancer patients and more than 600,000 deaths (2). So far, we still lack effective means to completely cure tumors. The occurrence and progression of tumor are accompanied by considerable mutations in oncogene and tumor suppressor genes (3). With in-depth studies on tumor genomics in recent years, diagnostic and therapeutic targets have been increasingly discovered (4). For example, biomarkers such as CEA and AFP have shown important value in tumor diagnosis, and biomarkers such as HER2 have provided effective direction for targeted therapy (5, 6). However, our understanding of the underlying mechanism of cancer is far from comprehensive, and it is highly necessary to discover more effective tumor markers.

Immune escape is one of the most important hallmarks for tumorgenesis and cancer development (7). Tumor cells evade immune surveillance through affecting their crosstalk with immune cells in tumor microenvironment (TME) (8). Tumor cells can abnormally express immunosuppressive checkpoints, interfere with specific antigen presentation, and secrete immunochemokines to directly influence the function of immune cells (9–11). In addition, abnormal metabolic status of tumor cells can also alter the composition of local TME, such as increasing lactic acid (12). The nutrient deficient acidic TME could weaken the function of immune cells such as CD8^+^ T cell (13). With the development of single-cell sequencing technology, the immunosuppressive microenvironment within tumors is increasingly understood. For instance, the detailed intratumoral heterogeneity and immunosuppressive TME in liver and brain metastases of breast cancer were revealed by using single cell sequencing (14). In recent years, immunotherapy based on immunocheckpoint inhibitors (ICIs) has been applied in clinical practice in the treatment of a variety of tumors (15). However, the response rate of tumor cells to immunotherapy remains low (16). Therefore, exploring the potential mechanism of tumor immune escape and screening more effective immune checkpoints has great significance in improving the efficiency of tumor immunotherapy. In addition to immunotherapy based on ICIs, new biotechnologies like molecular mimicry and cancer vaccine have also brought more options for cancer treatment (17).

Cadherin 5 (CDH5), also known as VE-cadherin, is one of the superfamily of transmembrane cadherin (CDH) proteins (18). CDH5 plays an important role in cell-cell adhesion that could maintain the integrity of endothelial and vascular tissues (18, 19). Previous study reported abnormal expression of CDH5 in different tumors, and that CDH5 could regulate tumor development by influencing angiogenesis (20–22). In glioma and melanoma, CDH5 stimulated tumor progression through inducing vascular formation (20, 21). In addition, a high expression of CDH5 is associated with a worse clinical outcome of patients with breast cancer and gastric cancer (22–24). However, the regulatory role of CDH5 in tumor immune escape and local immune response is rarely studied. In the present research, we investigated the correlation between CDH5 and the clinical features of cancer patients. The associations between CDH5 and the tumor immune response including immune cells infiltration, immunomodulators expression and chemokines expression were also analyzed. The results showed that CDH5 influenced the prognosis of many tumors and promoted the immune response in most tumors. Further experimental verification indicated that CDH5 was low-expressed in bladder cancer (BCa) and this was positively correlated with a better prognosis of BCa patients. The immune function assays results demonstrated that CDH5 could increase the immune response of BCa to CD8^+^ T cells. This current study provided a new target for tumor immunotherapy.

## Methods

### Data collection and processing

RNA sequencing data, clinical data, and mutation data in The Cancer Genome Atlas (TCGA) were obtained from the UCSC Xena (https://xena.ucsc.edu/). CDH5 expression data were extracted from the obtained data sets using Strawberry Perl (Version 5.32.0, http://strawberryperl.com/). Further data processing and analysis were performed using R software (Version 4.0.2; https://www.Rproject.org).

### Differential expression analysis

The expression level of CDH5 in 24 normal tissues and 33 tumor tissues were analyzed. The data was log2-transformed and the CDH5 expression was compared in tumor tissues and relative normal tissues. T test was applied to analyze the expression difference.

### Clinical significance analysis

The correlations between CDH5 and stage of each tumor type were analyzed. The associations between CDH5 and overall survival (OS), disease free survival (DFS), disease specific survival (DSS), and progression-free survival (PFS) were analyzed. The Kaplan-Meier curve was plotted for prognosis analysis for each tumor type.

### Tumor mutation burden and microsatellite instability analysis

The correlations between CDH5 and TMB or MSI of all tumor types were analyzed using Spearman’s rank correlation coefficient. The TMB and MSI data were calculated according to the mutation data.

### Gene set enrichment analysis

GSEA of CDH5 with all genes was conducted based on the TCGA data. Kyoto Encyclopedia of Genes and Genomes (KEGG) set based GSEA analysis and Hallmark set- based GSEA analysis of all types of tumor were conducted. Five enrichment pathways with the highest correlation were shown in the results.

### Correlation between CDH5 and immune response

Estimation of Stromal and Immune Cells in Malignant Tumor Tissues Using Expression Data (ESTIMATE) is an algorithm for evaluating the immune and stromal scores of tumors. The correlation between CDH5 and immune score in each tumor type was analyzed by using R software.

CIBERSORT, a metagene tool, was used to evaluate the infiltration scores of 26 types of immunocytes in each tumor. Associations between CDH5 and immune cell infiltration scores were calculated in R software.

In addition, the correlations between CDH5 and lymphocyte infiltration, immunoinhibitor, immunostimulator, MHC molecule, chemokine, and chemokine receptor in each tumor were analyzed using the TISIDB website (http://cis.hku.hk/TISIDB/).

### Patient tissues and cell lines

The BCa tissues and adjacent normal tissues (40) were acquired from patients undergoing radical surgery for BCa at the First Affiliated Hospital of Nanjing Medical University from 2016 to 2021. The informed consent was signed by all the patients. The use of tissues derived from BCa patients was approved by the Ethics Committee of The First Affiliated Hospital of Nanjing Medical University. The tissue was diagnosed as BCa tumor tissue via pathologically confirming, while the adjacent normal tissue was confirmed through pathologically diagnosing without tumor tissue. The BCa cell lines (T24, UMUC3, 253J, 5637, J82, BIU87, and RT4) and normal urothelial cell line (SV-HUC) were purchased from the Type Culture Collection of the Chinese Academy of Sciences (Shanghai, China).

### Cell culture and transfection

T24 cells were cultured at constant temperature of 37°C with 5% CO_2_ in an incubator with DMEM medium (Gibco, USA) containing 10% fetal bovine serum (BI, Israel). The overexpression plasmid of CDH5 and control vector were obtained from GenePharma (GenePharma, Shanghai, China). T24 cells were grew into 50% density in a six-well plate and transfected by CDH5 overexpression plasmid or control vector using the Lipofectamine 3000 kit (Invitrogen, USA).

### RNA isolation and quantitative real-time PCR

The BCa tissues or cells RNA were extracted by TRIzol reagent (Invitrogen, USA). After the concentration had been determined by the microplate reader (Tecan, Switzerland), RNA was reverse-transcribed into cDNA by the HiScript II reagent (Vazyme, China). The reaction system used in qRT-PCR experiment was prepared by the SYBR pre-mix kit (Vazyme, Nanjing, China). The StepOne Plus real-time PCR system (Applied Biosystems, USA) was used to conduct the qRT-PCR assay. The target RNA CT values were normalized via subtracting the CT values of β-Actin. The primers used in this study were acquired from TsingKe (**Table S1**).

### Western blot and immunohistochemistry

The tissues protein were extracting by using the RIPA buffer (Sigma, USA). Then we detected the concentration of extracting protein by using bicinchoninic acid (BCA) assays (Beyotime, China). After isolating by SDS-PAGE, proteins were transferred to PVDF membrane (Millipore, USA). After blocking by 5% skim milk, the PVDF membrane were incubated with CDH5 primary antibody (Protech, USA) and Goat anti-rabbit secondary antibody (Protech, USA). The expression level of CDH5 was detected by using Chemiluminescence (Bio-Rad, USA). The paraffin-embedded BCa tissues were sliced into 4 mm slides. After rehydrated by using different grades of ethanol, the antigens of slides were isolated by microwave heating. After dipping with 3% H_2_O_2_. the slides were treating with CDH5 primary antibody (Protech, USA) at 4°C for 12 hours. Then the slides were incubating with HRP-conjugated secondary antibody (Protech, USA). The results were observed and collected by using microscope. The expression level of CDH5 was confirmed by at least two pathologists.

### Screening, activating, and culturing of CD8^+^ T cell

The peripheral blood mononuclear cells (PBMCs) were separated from the peripheral blood of healthy persons using the PBMC separation reagent (FACs, Nanjing, China). Magnetic separation based on CD8 microbeads (Miltenyi, Germany) was conducted to screen CD8^+^ T cells from the PBMCs. CD8^+^ T cells were cultured in RPMI-1640 medium (Gibco, USA) and then activated by treating with CD3 antibodies (2 μg/mL; Invitrogen, USA), CD28 antibodies (1μg/mL; Invitrogen, USA), and interleukin 2 (IL-2, 5 ng/mL; R&D Systems, USA) for 72 hours.

### CD8^+^ T cell-BCa cell co-culture and CD8^+^ T cell functional assay

The BCa cells were co-cultured with activated CD8^+^ T cells at a ratio of 2.5:1 for 48 hours. Then the medium of co-culture system was collected and we performed ELISA assays to detect the content of IFN-γ and granzyme B using commercial kits (FACs, Nanjing, China). In addition, the BCa cells were co-cultured with the activated CD8^+^ T cells at a ratio of 1:2 for 72 hours. After removing the CD8^+^ T cells and cell debris from the system, the cells were washed with PBS for several times. The remaining living BCa cells were detected using a microplate reader at 570 OD. Afterwards, the remaining BCa cells were fixed by 4% paraformaldehyde and then stained with 0.1% crystal violet. The methods for CD8^+^ T cell screening, culturing, activating, and immune functional assays were reported in our previous research (25).

## Results

### Pan-cancer expression analysis of CDH5

**Figure 1:**
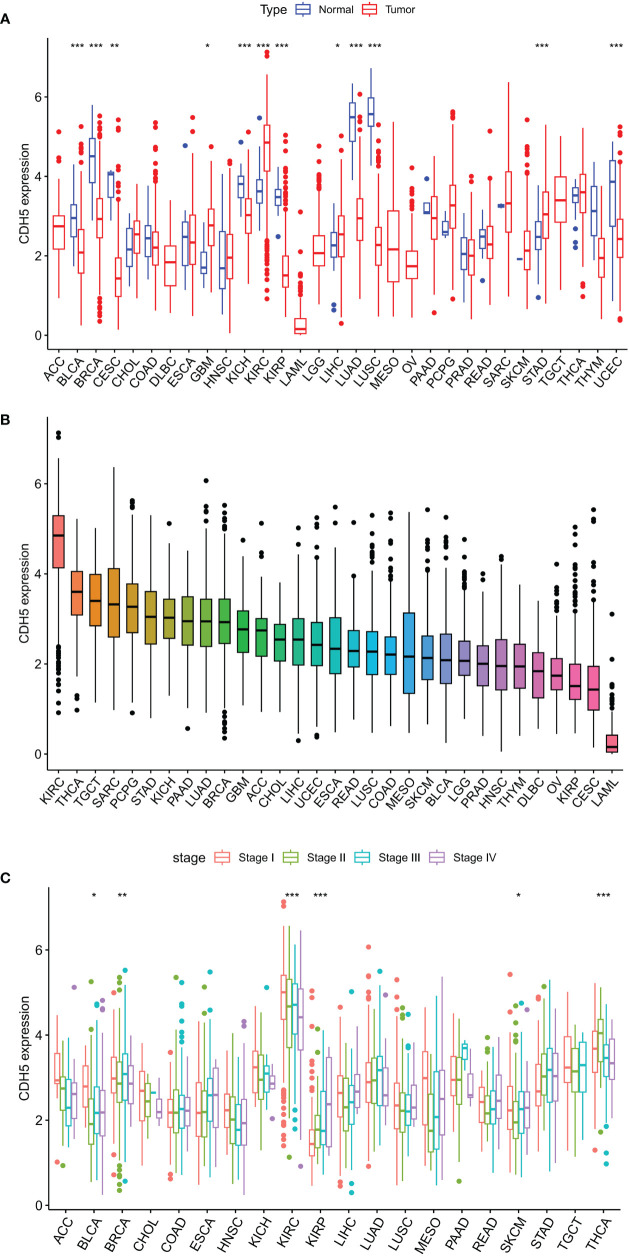
Differential expression analysis of CDH5 in TCGA pan-cancer. **(A)** The expression of CDH5 in TCGA pan-cancers and relative normal tissues (*P<0.05, **P<0.01, ***P<0.001, Student’s t-test). **(B)** The expression of CDH5 in 31 types of tumor. **(C)** Pan-cancer expression of CDH5 in different tumor stages (*P<0.05, **P<0.01, ***P<0.001, Student’s t-test).

The expression of CDH5 in TCGA pan-cancer was determined. The research process of this subject is shown in **Figure S1**. CDH5 was down-regulated in 8 tumor types of BLCA, BRCA, CESC, KICH, KIRP, LUAD, LUSC, and UCEC in comparison with relative normal tissues and high-expressed in 4 tumor types of GBM, KIRC, LIHC, and STAD in comparison with relative normal tissues (**Figure 1A**). The expression of CDH5 was compared in different tumor types, and CDH5 showed a high expression level in KIRC, THCA, and TGCT and a low expression in LAML, CESC, and KIRP (**Figure 1B**). We then detected the expression of CDH5 across different World Health Organization cancer stages, and found that CDH5 was high-expressed in stage I of BLCA, BRCA, KIRC, and SKCM when compared with higher stages but it was low-expressed in stage I of KIRP and THCA (**Figure 1C**).

### Prognosis analysis of CDH5 in pan-cancer

**Figure 2:**
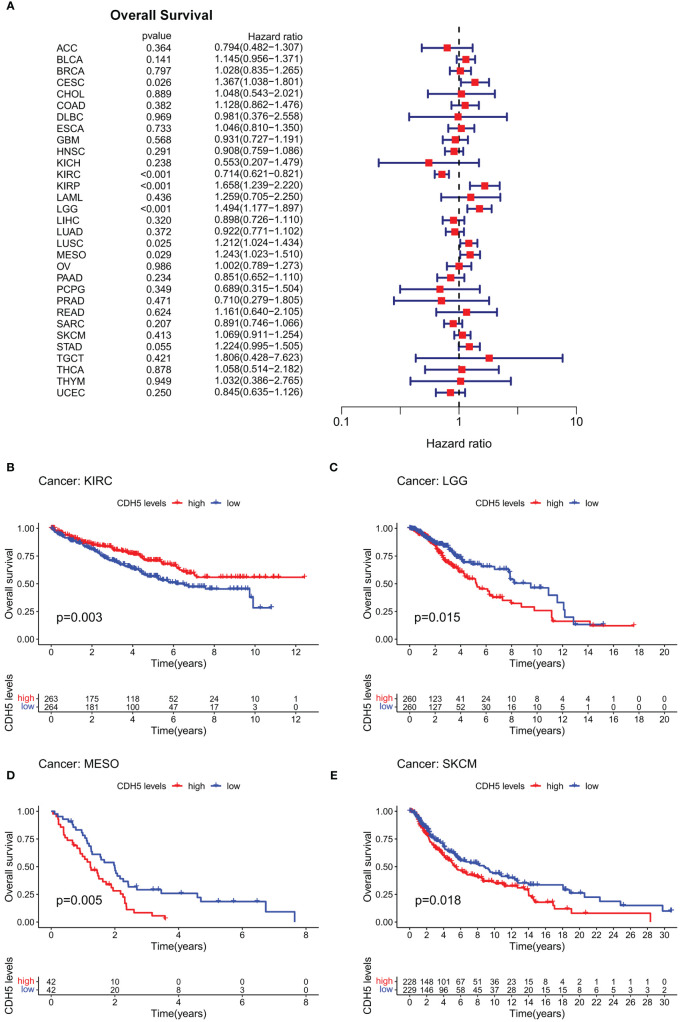
Correlation of CDH5 with overall survival time of TCGA pan-cancer. **(A)** Cox proportional hazards model of CDH5 in overall survival of TCGA pan-cancer. **(B-E)** Kaplan-Meier analysis of correlation between CDH5 and overall survival in different tumors.

In order to investigate the role of CDH5 in tumor prognosis, we conducted prognosis correlation analyses including OS, DFS, DSS, and PFS in pan-cancer. The result of Cox proportional hazards model analyses showed that CDH5 was significantly correlated with OS of CESC, KIRC, KIRP, LGG, LUSC, and MESO. It was a high-risk gene in CESC, KIRP, LGG, LUSC, and MESO, but a low-risk gene in KIRC (**Figure 2A**). The results of Kaplan-Meier survival analyses showed that a high expression of CHD5 was positively associated with a better OS of KIRC patients but negatively associated with a better OS of LGG, MESO, and SKCM patients (**Figures 2B-E**). Furthermore, Cox proportional hazards model analysis based on DFS demonstrated that CDH5 was significantly associated with DFS in CESC, KIRP, and UCEC. CDH5 was a high-risk gene in CESC and KIRP, but it was a low-risk gene in UCEC (**Figure S2A**). Kaplan-Meier survival analysis results demonstrated that high-expressed CHD5 was positively associated with a better DFS of LUAD and UCEC patients, while CDH5 was positively associated with a worse DFS of CESC patients (**Figures S2B-D**). In addition, Cox proportional hazards model analysis based on DSS indicated that CDH5 was significantly associated with DSS in CESC, KIRC, KIRP, LGG, and UCEC. It was a high-risk gene in CESC, KIRP, and LGG but a low-risk gene in KIRC and UCEC (**Figure S3A**). Kaplan-Meier survival analysis results showed that high-expressed CHD5 was positively associated with a better DSS of KIRC patients but it was positively associated with a worse DSS of KIRP, LGG, and SKCM patients (**Figures S3B-E**). Moreover, Cox proportional hazards model analysis based on PFS indicated that CDH5 was significantly associated with PFS in CESC, KIRC, KIRP, THCA, and UCEC. It was a high-risk gene in CESC and KIRP but a low-risk gene in KIRC, THCA, and UCEC (**Figure S4A**). Kaplan-Meier survival analysis results showed a high expression of CHD5 was positively associated with better PFS of HNSC, KIRC, and THCA patients, while CDH5 was positively associated with a worse PFS of SKCM patients (**Figures S4B-E**).

### Correlation analysis of CDH5 with TMB and MSI in pan-cancer

**Figure 3:**
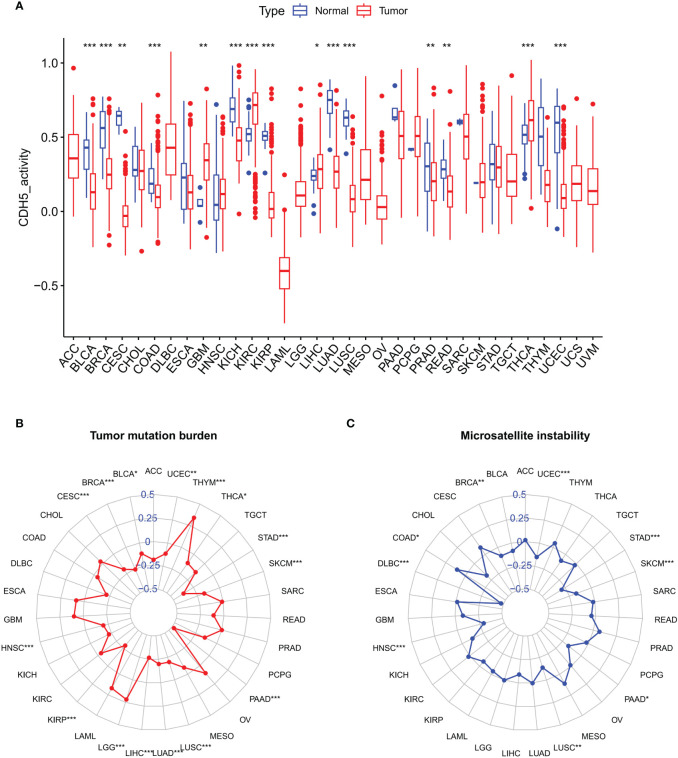
Associations between CDH and Tumor Mutation Burden (TMB) or Tumor Microsatellite Instability (MSI) in TCGA pan-cancer. **(A)** The CDH5 gene activity in TCGA pan-cancers (*P<0.05, **P<0.01, ***P<0.001, Student’s t-test). **(B)** The correlation between CDH5 and TMB in TCGA pan-cancers (*P<0.05, **P<0.01, ***P<0.001, Student’s t-test). **(C)** The correlation between CDH5 and MSI in TCGA pan-cancers (*P<0.05, **P<0.01, ***P<0.001, Student’s t-test).

We further studied the association between CDH5 and TMB or MSI in pan-cancer, which all showed a significant relationship with the sensitivity of ICIs. The gene activity of CDH5 in different tumors and relative normal tissues were first assess and the results indicated that the activity of CDH5 was lower in tumors including BLCA, BRCA, CESC, COAD, KICH, KIRP, LUAD, LUSC, PRAD, READ, and UCEC but it was higher in tumors including GBM, KIRC, LIHC, and THCA (**Figure 3A**). CDH5 was significantly associated with TMB in 15 tumors including UCEC, THYM, THCA, STAD, SKCM, PAAD, LUSC, LUAD, LIHC, LGG, KIRP, HNSC, CESC, BLCA, and BRCA (**Figure 3B**). And CDH5 was significantly associated with MSI in 9 tumors including UCEC, STAD, SKCM, PAAD, LUSC, HNSC, DLBC, COAD, and BRCA (**Figure 3C**).

### GSEA analyses of CDH5

**Figure 4:**
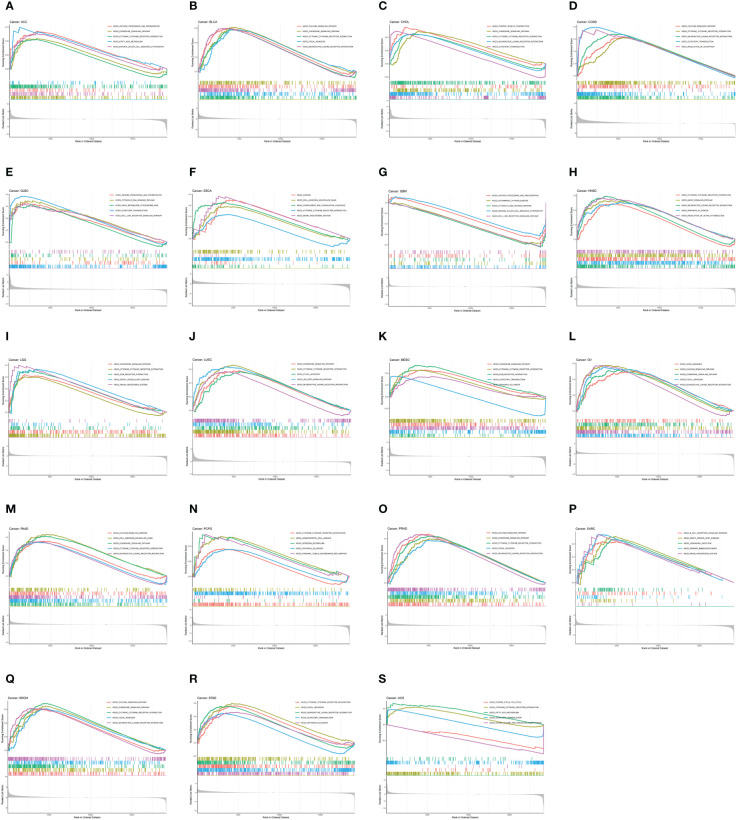
KEGG-GSEA of CDH5 in TCGA pan-cancer. **(A-S)** KEGG pathway analyses of CDH5 in different types of tumor. The results which was enriched in immune response pathway has been screened out.

**Figure 5:**
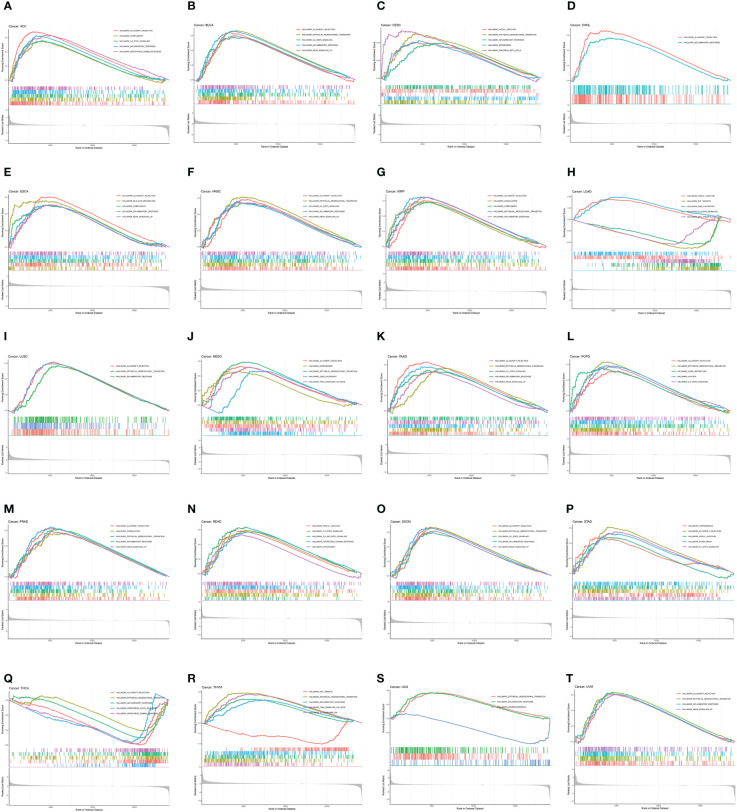
Hallmarks-GSEA of CDH5 in TCGA pan-cancer. **(A-T)** Hallmarks pathway analyses of CDH5 in different types of tumor. The results which was enriched in immune response pathway has been screened out.

To investigate the biological function of CDH5 in different types of tumor, KEGG-GSEA and Hallmarks-GSEA analyses were conducted. The KEGG pathway gene sets and Hallmark pathway gene sets were downloaded from the website (https://www.gsea-msigdb.org/gsea/index.jsp). KEGG-GSEA results showed that CDH5 was significantly correlated with immune regulative pathways such as antigen processing and presentation, chemokine signaling, cytokine-cytokine receptor interaction, natural killer cell mediated cytotoxicity, B cell receptor signaling, and primary immunodeficiency in 19 tumors including BLCA (**Figure 4**). Hallmarks-GSEA results demonstrated that CDH5 was significantly associated with immune regulative pathways such as complement, IL2-STAT5 signaling, inflammatory response, interferon gamma response, TNFA signaling via NFkB, IL6-JAK-STAT3 signaling, and interferon alpha response in 20 tumors including BLCA (**Figure 5**).

### Relationship between CDH5 and immune response in pan-cancer

**Figure 6:**
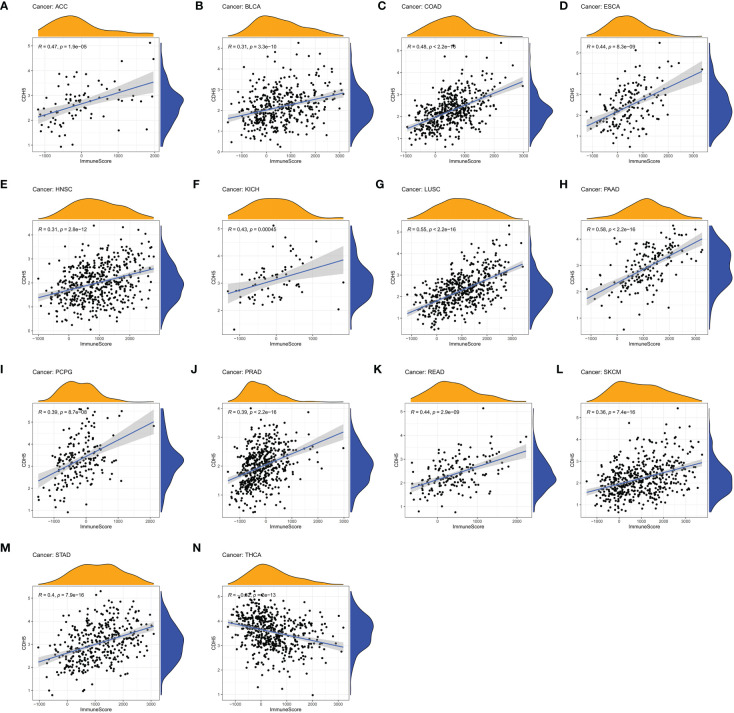
Correlation coefficients of CDH5 and immune scores of TCGA pan-cancer. **(A-N)** The associations between CDH5 and immune scores in different types of tumor.

**Figure 7:**
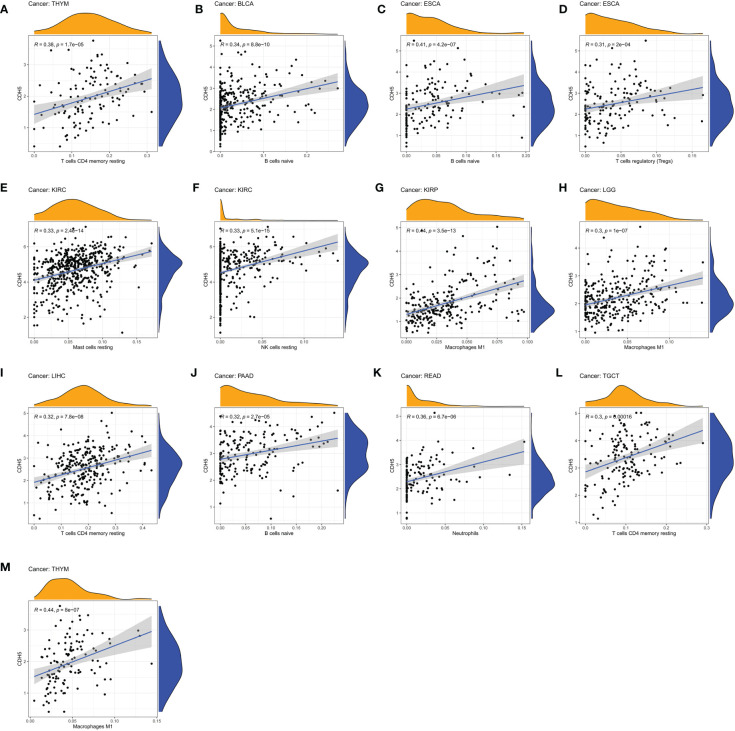
Associations between CDH5 and different immune cells infiltration in TCGA pan-cancer. **(A–M)** The associations between CDH5 and immune cells infiltration in different types of tumor.

**Figure 8:**
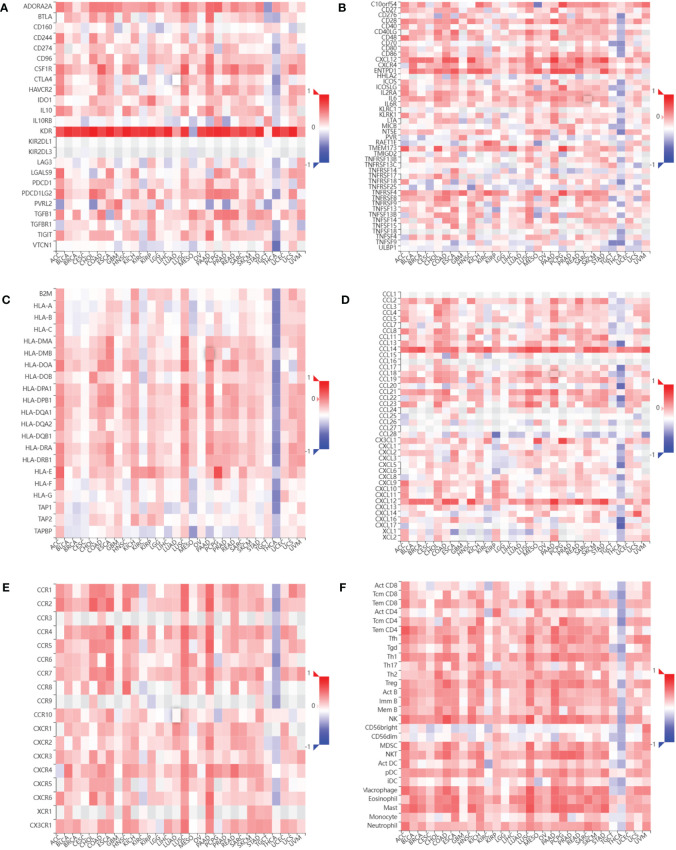
Relationship between CDH5 and immune response related genes. **(A)** The associations between CDH5 and immunoinhibitors in TCGA pan-cancers. **(B)** The associations between CDH5 and immunostimulators in TCGA pan-cancers. **(C)** The associations between CDH5 and MHC molecules in TCGA pan-cancers. **(D)** The associations between CDH5 and chemokines in TCGA pan-cancers. **(E)** The associations between CDH5 and chemokine receptors in TCGA pan-cancers. **(F)** The associations between CDH5 and infiltrated immune cells in TCGA pan-cancers.

We assessed the relationship between CDH5 expression and immune cells infiltration in tumors. The ESTIMATE algorithm was used to calculate the immune scores in pan-cancer, and the results showed that CDH5 was positively correlated with the immune scores of 13 tumors including ACC, BLCA, COAD, ESCA, HNSC, KICH, LUSC, PAAD, PCPG, PRAD, READ, SKCM, and STAD but CDH5 was negatively correlated with immune score of THCA (**Figure 6**). The stromal scores were also investigated in pan-cancer. The results showed CDH5 was positively associated with stromal score in 14 tumors including ACC, BLCA, COAD, ESCA, HNSC, KICH, LUSC, PAAD, PCPG, PRAD, READ, SKCM, STAD and THYM (**Figure S5**). CIBERSORT was used to analyze the correlations between CDH5 and different immune cell infiltration in pan-cancer. We found that CDH5 was positively associated with memory T cells, B cells, T regulatory cells, mast cells, NK cells, macrophages, and neutrophils infiltration in 13 tumors (**Figure 7**). In addition, we also performed the co-expression analyses to further study the associations between CDH5 expression and immune response genes in pan-cancer. The immune response genes consisted of lymphocytes infiltration relative genes, immunoinhibitors, immunostimulators, MHC molecules, chemokines, and chemokine receptors. The co-expression analyses results showed that almost all the immune response genes were positively associated with CDH5 in pan-cancers except THCA (**Figure 8**). We detected the correlation between CDH5 and real-world immunotherapy response by using BEST website (https://rookieutopia.com/). In Riaz 2018 immunotherapy cohort, patients with high expression of CDH5 had significant immunotherapy response (**Figure S6**). However, in Kim 2019 cohort, patients with high CDH5 expression had a worse immunotherapy response rate (**Figure S6**). The role of CDH5 in single-cell RNA-seq analyses was also investigated by using TISCH website (http://tisch.comp-genomics.org/). The results showed that CDH5 was mainly enriched in endothelial cells (**Figure S7**).

### CDH5 was down-regulated in BCa tissues and cell lines and was associated with a better prognosis

**Figure 9:**
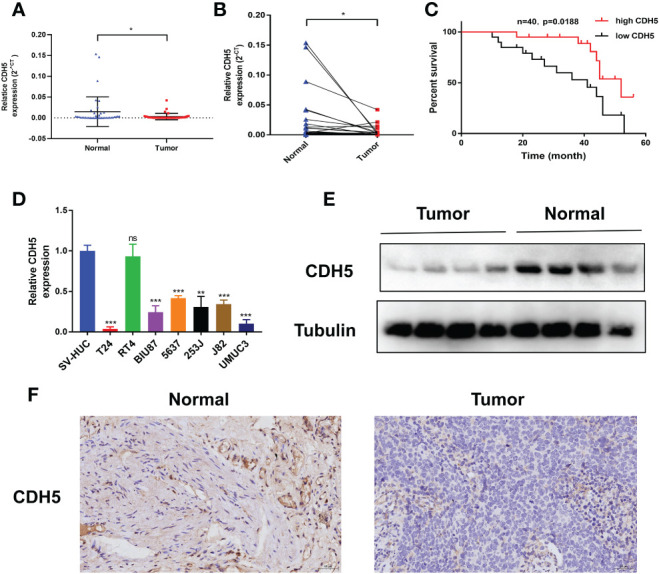
CDH5 was lowly expressed in BCa. **(A, B)** The CDH5 expression in 40 pairs of BCa tissues was validated by qRT-PCR (*P<0.05, Student’s t-test). **(C)** Kaplan-Meier analysis of correlation between CDH5 and overall survival of 40 BCa patients. **(D)** The expression of CDH5 in BCa cell lines and SV-HUC was validated by qRT-PCR (**P<0.01, ***P<0.001, Student’s t-test). **(E)** The expression of CDH5 in 4 pairs of BCa tissues was validated by western blot. **(F)** The expression of CDH5 in BCa tissues was validated by IHC. Data are mean ± SD, n=3.

The expression of CDH5 in 40 pairs of BCa tumors were detected by qRT-PCR. The results showed that CDH5 was low-expressed in BCa when compared with adjacent normal tissues (**Figures 9A, B**). Kaplan-Meier analysis demonstrated that patients with a higher expression of CDH5 had a better OS (**Figure 9C**). Furthermore, qRT-PCR results indicated that CDH5 was low-expressed in 6 BCa cell lines (T24, BIU87, 5637, 253J, J82, and UMUC3) when compared with SV-HUC (**Figure 9D**). Western blot results showed that CDH5 was highly expressed in adjacent normal tissues when compared with BCa tissues (**Figure 9E**). Results of IHC indicated that CDH5 was lowly expressed in BCa tissue when compared with adjacent normal tissue (**Figure 9F**).

### CDH5 promoted the anti-tumor ability of CD8^+^ T cells in BCa

**Figure 10:**
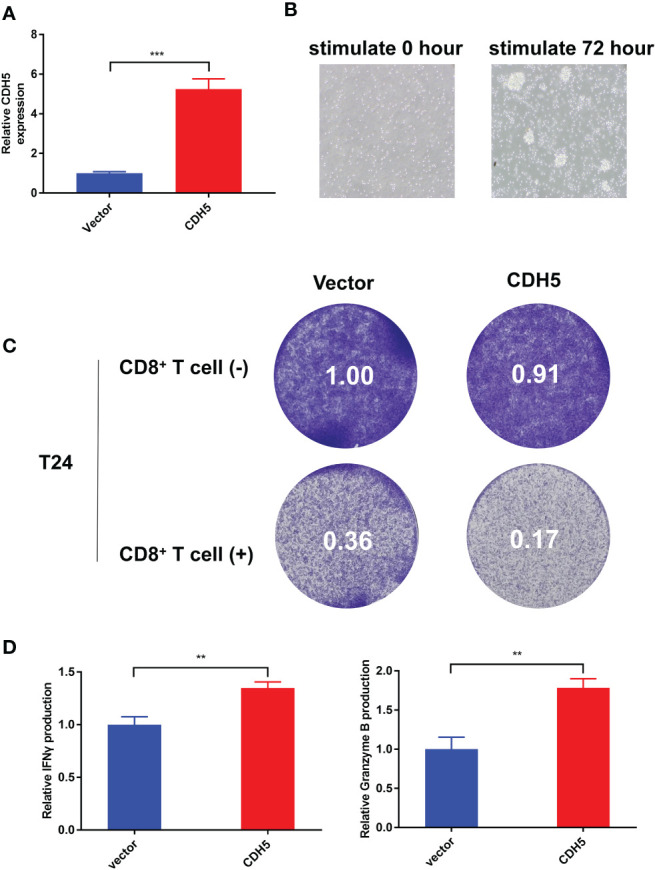
CDH5 promoted the function of CD8^+^ T cells in BCa. **(A)** The overexpression of CDH5 in T24 cells was validated by qRT-PCR (***P<0.001, Student’s t-test). **(B)** The microscope images of CD8^+^ T cells before and after activation. **(C)** Results of BCa-CD8^+^ T cell co-culture assay and CD8^+^ mediated tumor killing assay indicated the function of CD8^+^ T cells was promoted when co-cultured with CDH5 overexpressed BCa cells. **(D)** Results of ELISA assays indicated that CD8^+^ T cells produced more IFN-γ and granzyme B when co-cultured with CDH5 overexpressed BCa cells (**P<0.01, Student’s t-test). Data are mean ± SD, n=3.

According to our qRT-PCR result, the expression of CDH5 in T24 cells was the lowest among the 7 bladder cancer cell lines. Therefore, we selected T24 cells to construct CDH5-overexpressing BCa cells. The CDH5 over-expression and relative control plasmids were successfully transfected into T24 cells (**Figure 10A**). The CD8^+^ T cells were screened from the peripheral blood of healthy persons and then activated by CD3 antibody, CD28 antibody, and IL2. The activated CD8+ T cells showed obvious expansion and cluster growth (**Figure 10B**). The tumor cell killing ability of CD8^+^ T cells was promoted after co-culturing with CDH5 overexpressed T24 cells (**Figure 10C**). ELISA assays results indicated that CD8^+^ T cells produced more IFN-γ and granzyme B after co-culturing with CDH5 overexpressed T24 cells (**Figure 10D**). In conclusion, CDH5 could promote the function of CD8^+^ T cells in the TME of BCa.

## Discussion

In the last few years, a wide use of immunotherapy based on PD1/PDL1 and other ICIs has gradually provided new clinical treatment modalities, especially for patients with locally advanced or metastatic tumors who have lost the chance for taking surgery (26, 27). Although ICIs therapies have achieved tumor remission and prolonged patient survival, only a relatively small proportion of patients could benefit, which limits the clinical use of ICIs (28). Therefore, developing new immune checkpoint inhibitors to improve the efficacy and accuracy of treatment of ICIs has great clinical significance.

In recent years, more and more molecules or genes have been found to be related to the effects of tumor immunotherapy through pan-cancer analyses. For instance, cancer-associated fibroblast-derived biglycan was confirmed having great potential acting as therapeutic target in immunotherapy (29). In addition, RUNX Protein Family and FOXO Family were also confirmed having good correlation with tumor progression, clinical outcome and drug sensitivity by pan-cancer analyses (30, 31). CDH5 as a vascular endothelial transmembrane calmodulin plays an important role in the process of vasculogenic mimicry (32). Previous studies demonstrated that a high CDH5 expression is mainly associated with tumor angiogenesis (20, 21). The aberrant expression of CDH5 in a variety of tumor tissues has been verified by bioinformatics analysis and experiments, and the results confirmed that high-expressed CDH5 was positively correlated with worse survival and higher tumor stage (33). Hendrix et al. also reported that the down-regulation of CDH5 gene expression inhibits vasculogenic mimicry (20). However, our research verified that CDH5 was downregulated in urothelial carcinoma tissues and cell lines. Moreover, we also found that a low expression of CDH5 was associated with more advanced tumor stage and poorer prognosis of BCa. Therefore, we considered that there may be other ways to affect the etiology or pathogenesis of cancer, especially for BCa.

Our findings suggested that CDH5 played an important role in cancer immunity. Database analysis showed that CDH5 was closely associated with TMB, MSI, immune cell infiltration and immune response genes in a variety of tumors including BCa. This suggested that the CDH5 may affect the response of patients to ICIs therapy through influencing tumor immune response. Furthermore, KEGG enrichment analysis demonstrated that CDH5 and its co-expressed mRNA were enriched to some signaling pathways such as cytokine-cytokine receptor interaction-related signaling pathway, JAK-STAT signaling pathway, chemokine signaling pathway, IL2-STAT5 signaling pathway that have been reported to be associated to tumor immunity (34–38). These results indicated that the high expression of CDH5 in BCa can inhibit tumor growth through activating the immune activity of CD8^+^ T cells to kill tumor cells. The results of co-culture assay and ELISA experiments in BCa also showed that CDH5 could promote the function of CD8^+^T cells in BCa TME, which further confirmed that CDH5 can be used as a potential immune biomarker.

In our study, CDH5 was found to be positively correlated with immune cell scores in 25 tumor tissues, including BCa. Additionally, the correlation analysis revealed significant associations between CDH5 and immune cells (such as T cells, B cells, Macrophage cells, Treg cells, DC cells, and so on). In summary, our results confirmed that CDH5 was closely associated with tumor immune response, and that it could be used as a new immune checkpoint for immunotherapy in the future.

## Conclusions

Taken together, CDH5 was differentially expressed in many tumors and was significantly correlated to tumor prognosis. CDH5 was positively associated with tumor immunity and showed great potential to act as an immune checkpoint for cancer immunotherapy.

## Data availability statement

The original contributions presented in the study are included in the article/**Supplementary Material**. Further inquiries can be directed to the corresponding author.

## Ethics statement

All human-associated tissues used in this research were approved by the Ethics Committee of Nanjing Medical University. The studies were conducted in accordance with the local legislation and institutional requirements. The participants provided their written informed consent to participate in this study.

## Author contributions

JL, JWG and YL drafted the article and interpreted data. JWG, JL and QW performed the experiments and obtained the data. JL, JWG and YL designed the study. All authors contributed to the article and approved the submitted version.

## Supplementary material

Supplementary Figure 1 The research process of this subject.

Supplementary Figure 2 Correlation of CDH5 with disease free survival time of TCGA pan-cancer. **(A)** Cox proportional hazards model of CDH5 in disease free survival of TCGA pan-cancer. **(B-D)** Kaplan-Meier analysis of correlation between CDH5 and disease free survival in different tumors.

Supplementary Figure 3 Correlation of CDH5 with disease specific survival time of TCGA pan-cancer. **(A)** Cox proportional hazards model of CDH5 in disease specific survival of TCGA pan-cancer. **(B-E)** Kaplan-Meier analysis of correlation between CDH5 and disease specific survival in different tumors.

Supplementary Figure 4 Correlation of CDH5 with progression free survival time of TCGA pan-cancer. **(A)**. Cox proportional hazards model of CDH5 in progression free survival of TCGA pan-cancer. **(B-E)**. Kaplan-Meier analysis of correlation between CDH5 and progression free survival in different tumors.

Supplementary Figure 5 Correlation coefficients of CDH5 and stromal scores of TCGA pan-cancer. **(A-N)** The associations between CDH5 and stromal scores in different types of tumor.

Supplementary Figure 6 Relationship between CDH5 and immunotherapy response. **(A-Q)** The associations between CDH5 and immunotherapy response in different real-world immunology cohorts.

Supplementary Figure 7 Single-cell RNA sequencing data of CDH5 in pan-cancer.
