# Supplementary material for: A comprehensive pan-cancer analysis of CDH5 in immunological response
Source: Front Immunol. 2023 Sep 21;14:1239875. doi: 10.3389/fimmu.2023.1239875 (PMC10551168; doi:10.3389/fimmu.2023.1239875)
Supplement: Supplementary file 1 [file Table_1.doc]

**Table S1: All PCR primers used in this research**

| **Primers** |  | **sequence （5’-3’）** |
| --- | --- | --- |
| CDH5 | Forward  Reverse | 5’-TTGGAACCAGATGCACATTGAT-3’  5’-TCTTGCGACTCACGCTTGAC-3’ |
| β-Actin | Forward  Reverse | 5’-CTCCATCCTGGCCTCGCTGT-3’  5’-GCTGTCACCTTCACCGTTCC-3’ |
