# Supplementary figures and images for: A comprehensive pan-cancer analysis of CDH5 in immunological response
Source: Front Immunol. 2023 Sep 21;14:1239875. doi: 10.3389/fimmu.2023.1239875 (PMC10551168; doi:10.3389/fimmu.2023.1239875)

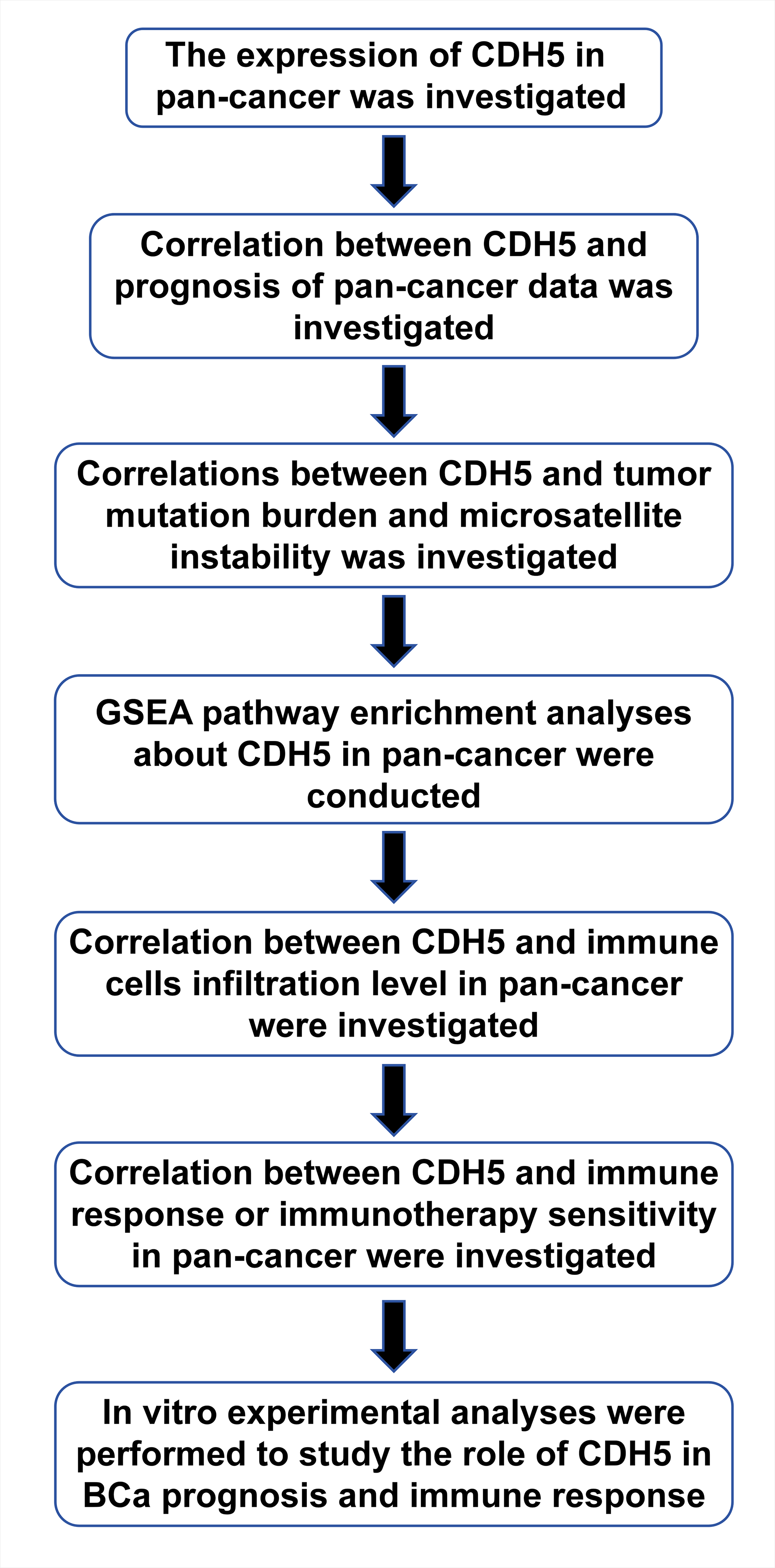

Supplement: Supplementary Figure 1 — The research process of this subject. [file Image_1.tif]

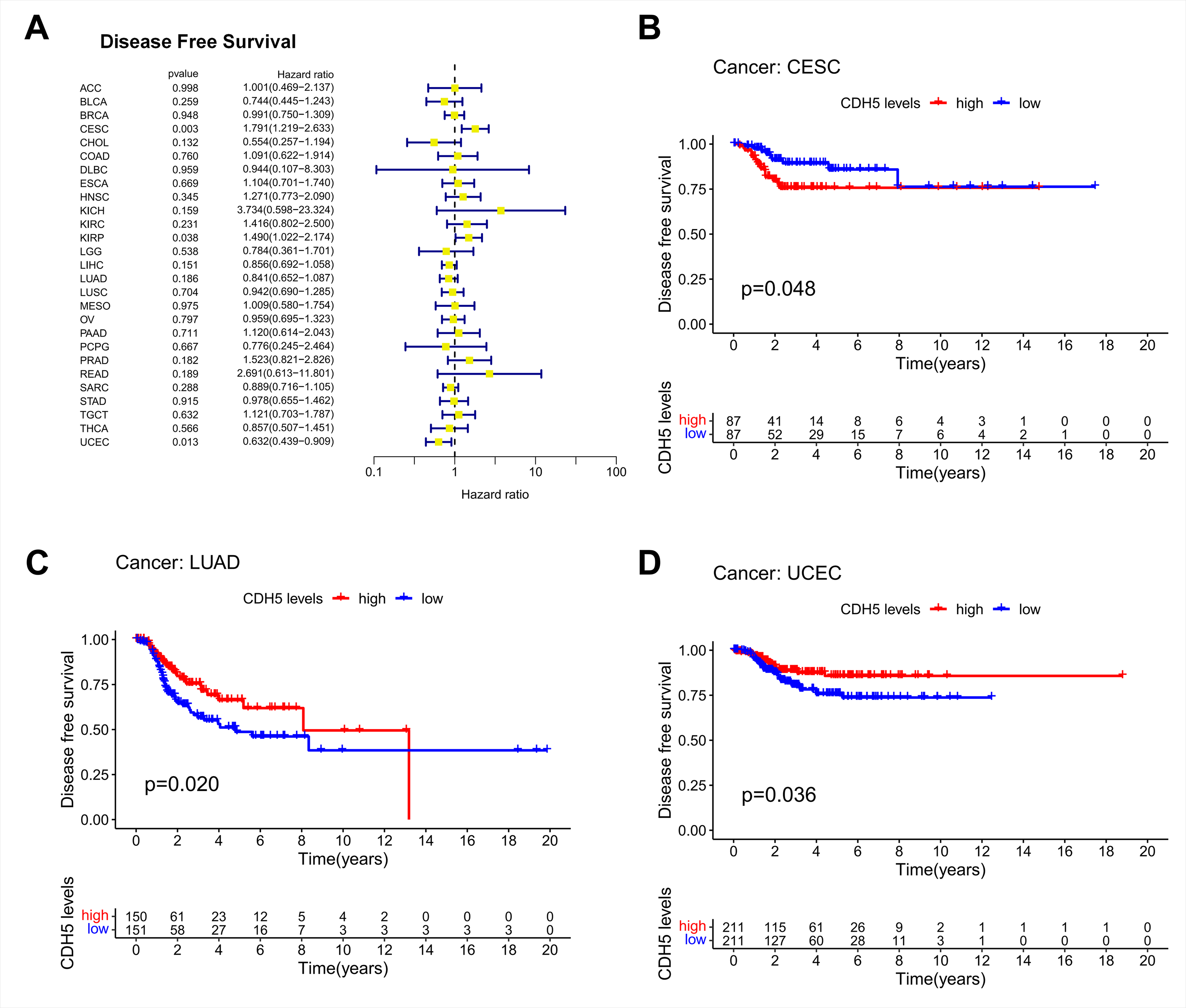

Supplement: Supplementary Figure 2 — Correlation of CDH5 with disease free survival time of TCGA pan-cancer. (A) Cox proportional hazards model of CDH5 in disease free survival of TCGA pan-cancer. (B-D) Kaplan-Meier analysis of correlation between CDH5 and disease free survival in different tumors. [file Image_2.tif]

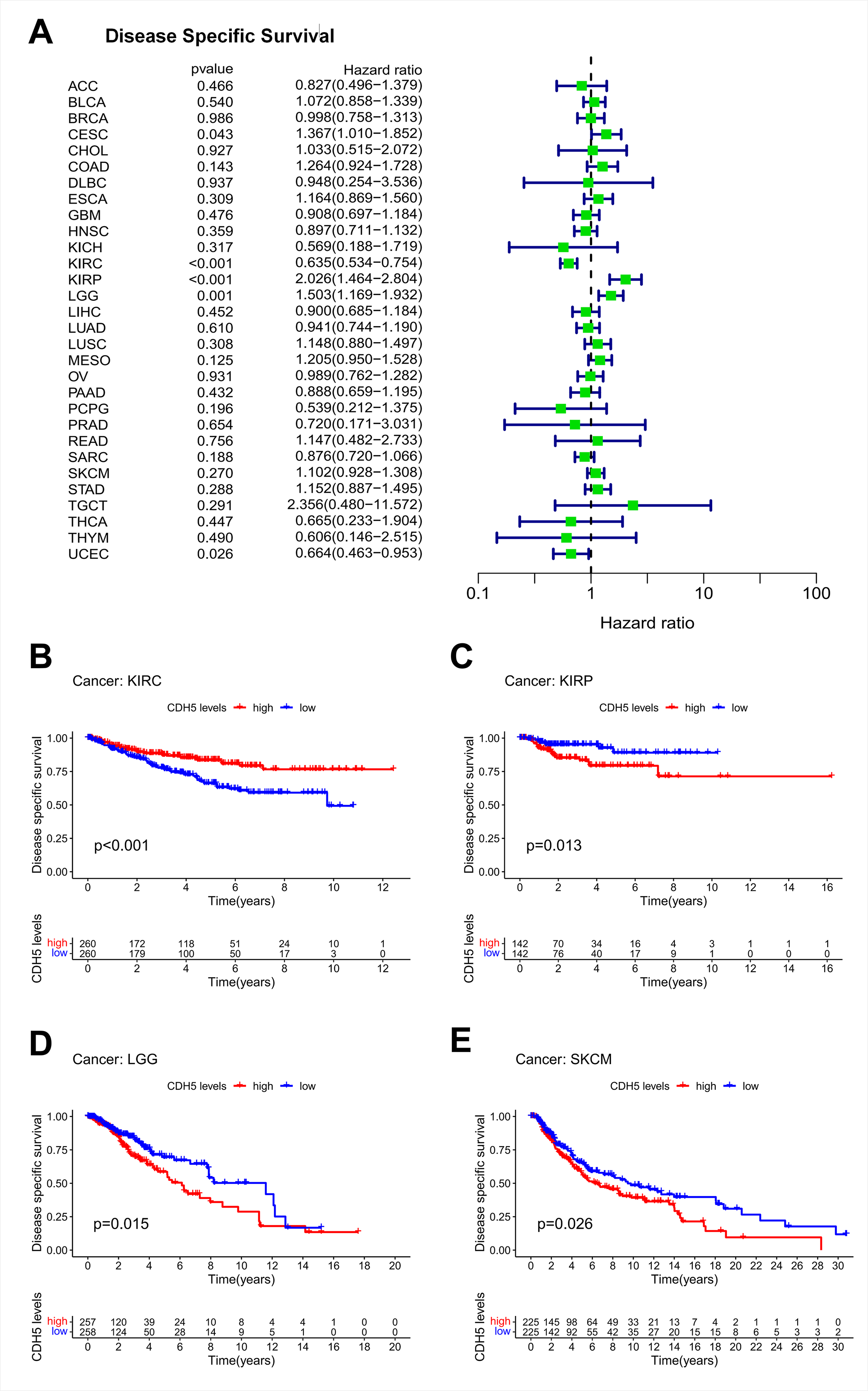

Supplement: Supplementary Figure 3 — Correlation of CDH5 with disease specific survival time of TCGA pan-cancer. (A) Cox proportional hazards model of CDH5 in disease specific survival of TCGA pan-cancer. (B-E) Kaplan-Meier analysis of correlation between CDH5 and disease specific survival in different tumors. [file Image_3.tif]

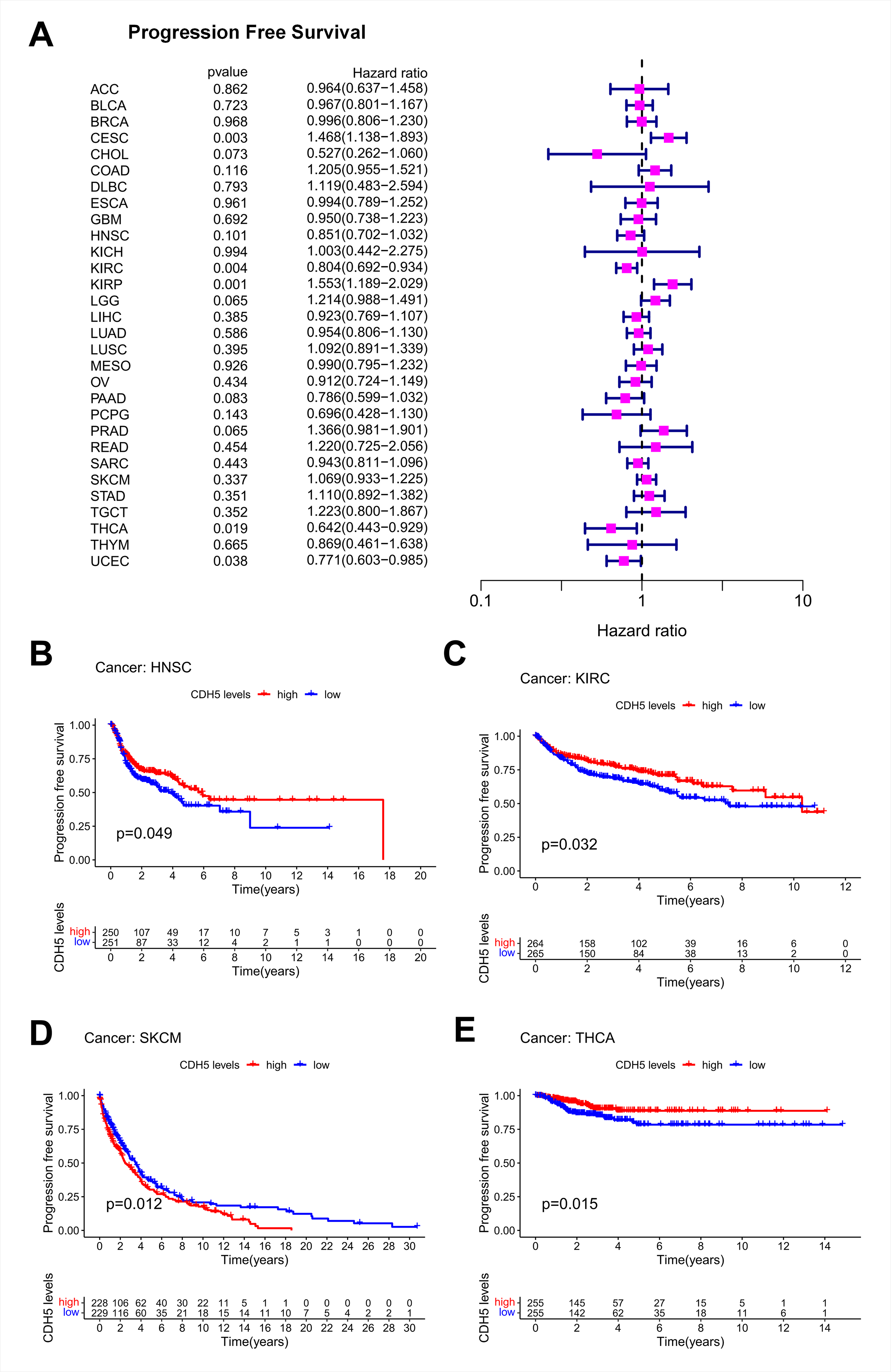

Supplement: Supplementary Figure 4 — Correlation of CDH5 with progression free survival time of TCGA pan-cancer. (A). Cox proportional hazards model of CDH5 in progression free survival of TCGA pan-cancer. (B-E). Kaplan-Meier analysis of correlation between CDH5 and progression free survival in different tumors. [file Image_4.tif]

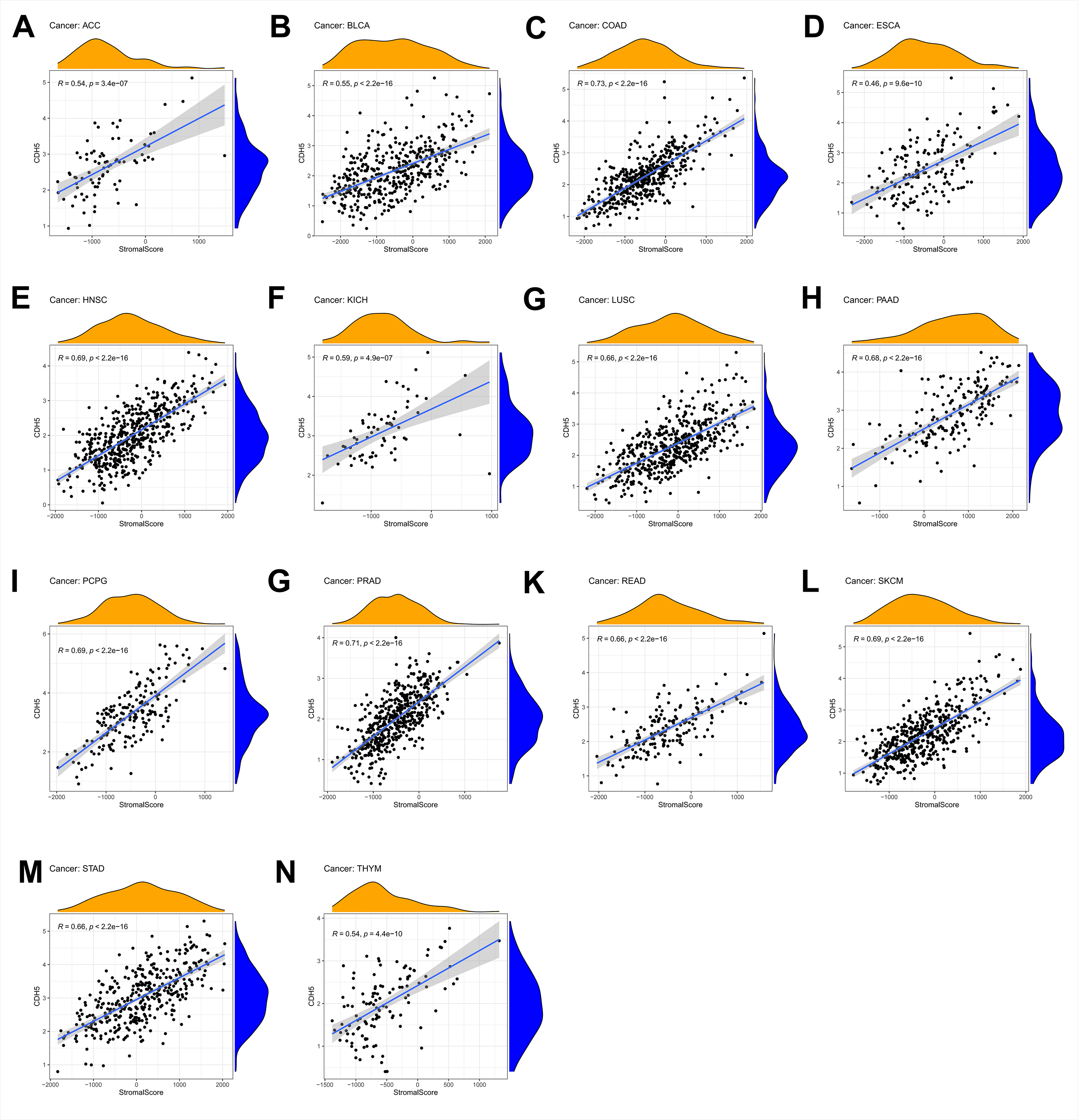

Supplement: Supplementary Figure 5 — Correlation coefficients of CDH5 and stromal scores of TCGA pan-cancer. (A-N) The associations between CDH5 and stromal scores in different types of tumor. [file Image_5.tif]

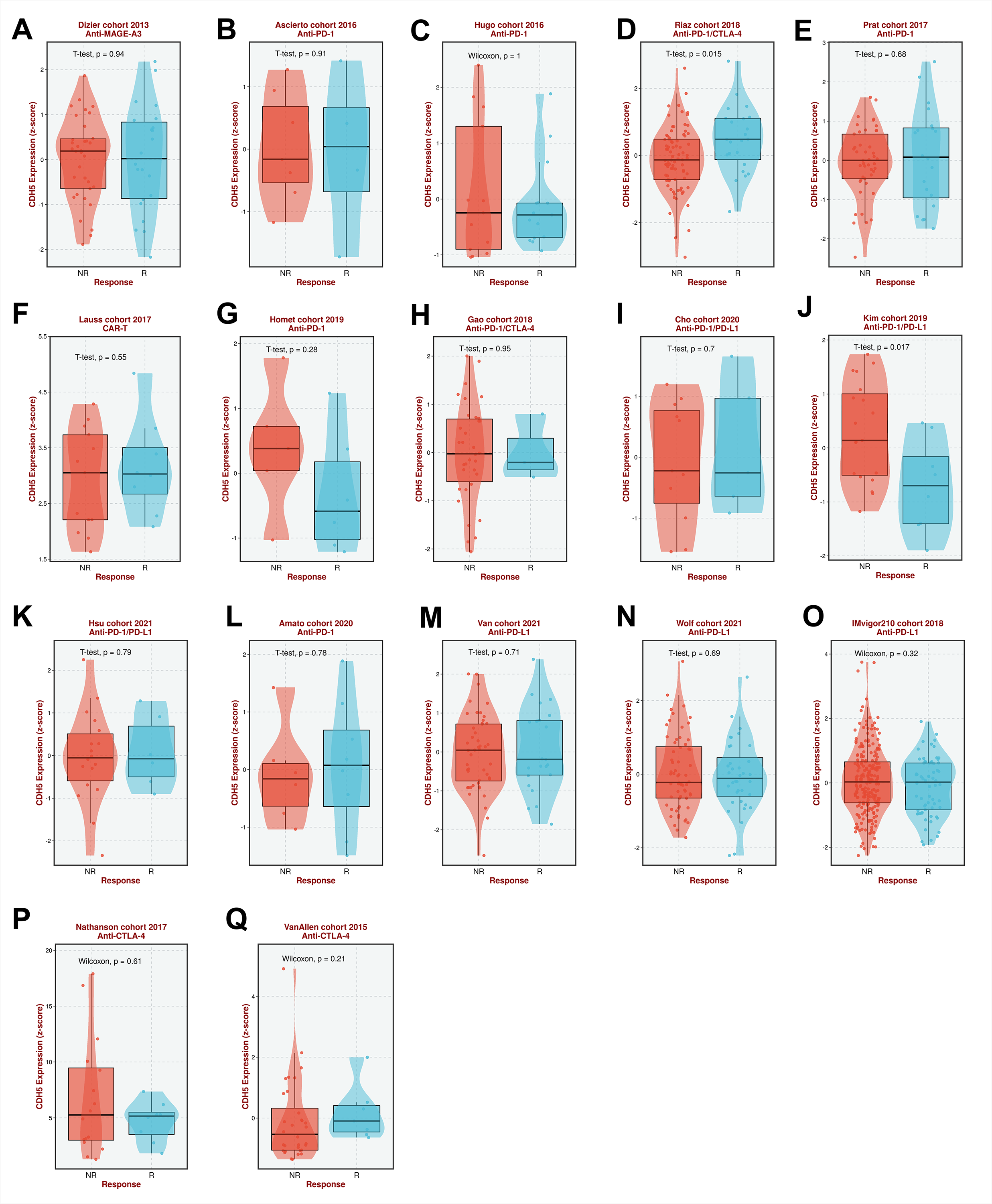

Supplement: Supplementary Figure 6 — Relationship between CDH5 and immunotherapy response. (A-Q) The associations between CDH5 and immunotherapy response in different real-world immunology cohorts. [file Image_6.tif]

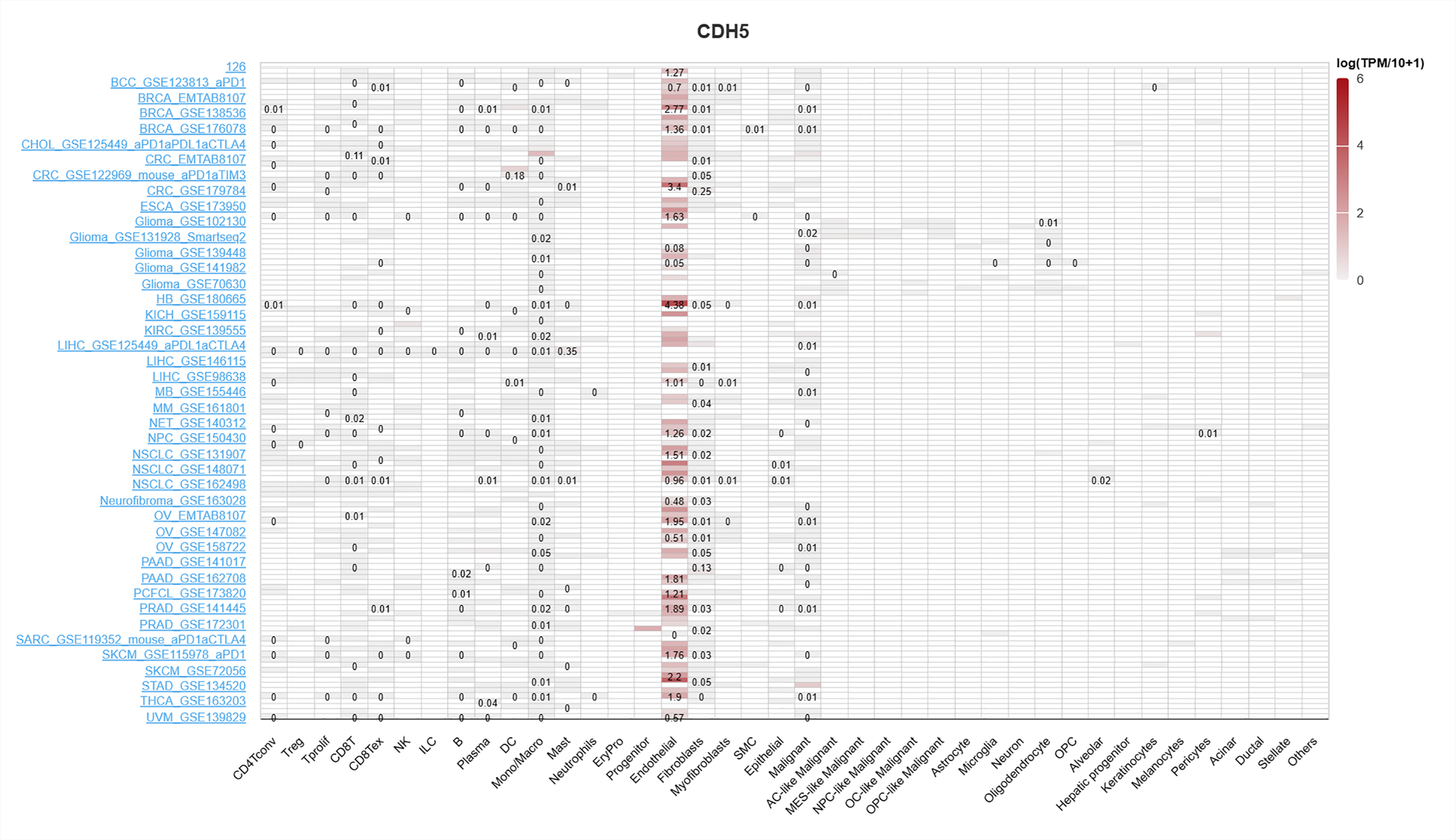

Supplement: Supplementary Figure 7 — Single-cell RNA sequencing data of CDH5 in pan-cancer. [file Image_7.tif]
